# Effects of prednisolone on 1,2‐O‐dilauryl‐rac‐glycero glutaric acid‐(60‐methylresorufin) ester‐lipase activity and pancreatic lipase immunoreactivity in healthy cats

**DOI:** 10.1111/jvim.17042

**Published:** 2024-03-14

**Authors:** Militsa Pacheva, Daniel Brugger, Barbara Riond, Matthias Dennler, Peter Hendrik Kook

**Affiliations:** ^1^ Clinic for Small Animal Internal Medicine, Vetsuisse Faculty University of Zurich Zurich Switzerland; ^2^ Institute of Animal Nutrition and Dietetics, Vetsuisse Faculty University of Zurich Zurich Switzerland; ^3^ Clinical Laboratory, Department for Clinical Diagnostics and Services, Vetsuisse Faculty University of Zurich Zurich Switzerland; ^4^ Clinic of Diagnostic Imaging Department of Clinical Services Vetsuisse Faculty University of Zurich Zurich Switzerland

**Keywords:** cat, corticosteroids, pancreatic enzymes, pancreatitis, triglyceride

## Abstract

**Background:**

Corticosteroids are among the most commonly used drugs in cats and are increasingly discussed as a treatment for feline pancreatitis. However, its effects on serum lipase in healthy cats remain unknown.

**Objectives:**

To evaluate the effects of prednisolone on serum lipase activity and pancreatic lipase immunoreactivity (PLI) in cats.

**Animals:**

Seven clinically healthy colony cats, aged 4 to 7 years, with unremarkable CBC/biochemistry panel were studied.

**Methods:**

Prospective study: Prednisolone (1.1‐1.5 mg/kg, median 1.28 mg/kg PO) was given daily for 7 consecutive days. Lipase activity (LIPC Roche; RI, 8‐26 U/L) and PLI (Spec fPL; RI, 0‐3.5 μg/L) were determined at day 1 before first treatment and at days 2, 3, 8, 10, and 14. Cats were examined daily. An a priori power analysis indicated that 6 cats were needed to find a biological relevant effect at 1‐β = 0.8. Statistical analyses comprised the Friedman test, random intercept regression, and repeated‐measures linear regression.

**Results:**

Median (range) day 1 lipase activities and PLI were 22 U/L (14‐52 U/L) and 3.2 μg/L (2.3‐15.7 μg/L). One cat with abnormally high lipase activity (52 U/L) and PLI (15.7 μg/L) at day 1 continued having elevated lipase activities and PLI throughout the study. Lipase activities and PLI concentrations did not differ significantly among time points regardless of whether the cat with elevated values was included or not. All cats remained healthy throughout the study.

**Conclusions and Clinical Importance:**

Administration of prednisolone in anti‐inflammatory doses does not significantly increase serum lipase activity and PLI concentration.

**Abbreviations**
DGGR: 1,2‐O‐dilauryl‐rac‐glycero glutaric acid‐(60‐methylresorufin) ester
PLI: pancreatic lipase immunoreactivity
RI: reference interval
SAA: serum amyloid A

## 1 INTRODUCTION

Histological evidence of pancreatitis is common in cats,^1^, ^2^ and pancreatitis is assumed a common clinical condition in cats, although prevalence data are lacking. Pancreatitis is almost always clinically diagnosed because histopathological confirmation of pancreatitis is highly invasive, offers no therapeutic advantage over a clinical diagnosis, and focal disease processes can be missed. Because clinical signs are often vague and nonspecific in cats with pancreatitis, and it can be difficult to ensure a complete ultrasonographic visualization of the entire pancreas, extra emphasis has been placed on lipase measurements in the last 15 years. Determination of serum lipase, either as a concentration (pancreatic lipase immunoreactivity [PLI]) or an activity (1,2‐o‐dilauryl‐rac‐glycero‐3‐glutaric acid‐(60‐methylresorufin) ester [DGGR]‐based lipase assays)^3^ has thus largely replaced histopathology as a surrogate gold standard for the diagnosis of pancreatitis in cats.^4^, ^5^, ^6^, ^7^, ^8^, ^9^ Both tests correlate strongly.^6^, ^8^ When compared with a standardized pancreatic histopathologic examination of the entire pancreas,^2^ or to a detailed pancreatic ultrasonographic examination,^8^ the LIPC Roche lipase assay and the PLI assay have comparable diagnostic sensitivity and specificity.^2^, ^8^ However because of the lack of a reliable and practicable gold standard, it is still unclear at what lipase cutoff pancreatitis is truly present or absent and whether measuring PLI or a DGGR‐based lipase activity makes a difference.

Corticosteroids are among the most frequently used drugs in cats. The effect of corticosteroids on various serum biochemistry panel results has been evaluated in healthy cats, however, lipase activity or PLI concentration was not included in these studies.^10^, ^11^, ^12^

Therefore, the aim of our study was to evaluate the effect of prednisolone on lipase activity and PLI concentration in healthy cats. Serum amyloid A (SAA), the most relevant acute phase protein in cats, was also measured to be as accurate as possible in monitoring inflammation in the event of steroid‐induced pancreatitis. Cholesterol and triglyceride concentrations were comeasured because hyperlipidemia has been implicated as both, a possible cause and consequence of steroid‐induced pancreatitis. We hypothesized that a 7‐day course of anti‐inflammatory doses of prednisolone would not affect lipase results.

## 2 MATERIALS AND METHODS

### 2.1 Animals

Seven clinically healthy cats from a research colony at the Vetsuisse faculty, University of Zurich were enrolled in our study. Cats were housed in an indoor and outdoor colony and daily supervised by certified technicians. None of the cats had been in an experiment in the last 18 months, and all cats had only been in feeding trials so far. All cats received their regular diet (Royal Canin Expert Adult Cat) fed ad libitum. Inclusion criteria were available documentation of stable body weights (recorded weekly) and absence of any health problem for the last 12 months before the study, an unremarkable clinical examination and normal CBC, and a biochemistry profile 1 week before the study.

The study was approved by the Cantonal Veterinary Office of Zurich and conducted in accordance with guidelines established by the Animal Welfare Act of Switzerland (No ZH195/2021).

### 2.2 Study design

This was a prospective longitudinal observational study. Prednisolone (Hedylon, 5 mg tablets, Graeub) in a median dose of 1.3 mg/kg (range, 1.1‐1.5 mg/kg) was administered PO in the morning for 7 consecutive days. Blood samples were collected on days 1, 2, 3, 8, 10, 14. Blood samples were always collected immediately before prednisolone administration, meaning that results from day (d) 1 are baseline values. All cats underwent daily physical examinations by 2 clinicians (resident and board‐certified internist) during the study and were weighed daily. Discontinuation criteria were defined as weight loss >5%, inappetence, vomiting, or diarrhea for more than 1 day. Blood samples were stored at room temperature and brought to the laboratory within 2 hours after collection. Serum lipase activity, cholesterol, and triglyceride concentrations were measured immediately at our institutional laboratory on Cobas. Serum amyloid A (Turbidimetric immunoassay MAST Eiken, Tokyo, Japan) was measured by an assay recently validated for use in cats.^13^ Lipase activity was measured by DGGR‐based assay (LIPC, Roche on Cobas, Roche Diagnostics, Rotkreuz, Switzerland; RI, 9‐26 U/L).^6^ Serum samples for PLI measurement (Spec fPL) were sent to IDEXX Diavet Switzerland. At the time of the study, the RI for PLI concentration was 0 to 3.5 μg/L. PLI concentrations of >5.4 μg/L were considered consistent with pancreatitis, and a concentration of 3.5 to 5.3 μg/L was considered equivocal. At the time of submitting the manuscript, the RI had been changed to 0 to 4.4 μg/L, values >8.8 μg/L are now interpreted as “consistent with pancreatitis,” whereas the equivocal range comprises 4.5 to 8.7 μg/L.^14^ No modifications had been made to the assay.

### 2.3 Statistical analysis

The minimum sample size for the present study was estimated with G*Power v3.1.9.7.^15^ Because no suitable cat data sets were identified, results from a dog study^16^ were used to estimate expected effect sizes of prednisolone treatment on serum lipase results over time. In dogs, a treatment‐dependent increase in pancreatic lipase by a factor of 2 ± 0.3 over 7 consecutive days was found,^16^ which corresponds to an effect size of *f* = 1.67, representing a strong statistical effect. It was estimated that a minimum number of 6 cats was needed to identify this effect as significant at *α* = 0.05 and 1‐*β* = 0.8. One additional cat was enrolled to account for potential study dropout. Data were analyzed by repeated‐measures mixed model analysis with SAS 9.4, applying the procedure mixed and including sex and time as fixed effects and the individual animal as random effect. Residuals were checked for normality via QQ‐Plots. If the precondition for normality was not met, a nonparametric approach (Friedman test) was applied.

The response of lipase activity and PLI concentration over time was plotted as random intercept mixed model analysis to highlight the data structure. In addition, the relationship between lipase activity (*x*) and PLI concentration (*y*) was analyzed by linear regression (*y* = *a* + *bx*), which was mapped by repeated‐measures mixed model analysis according to Shan et al^17^ applying the procedure MIXED. Thereby, the individual animal was considered as a random factor and “time” as repeating variable with the individual animal nested under the respective time point. A compound symmetry was mapped as suitable covariance matrix. The residuals of the model were successfully tested for normality by the Kolmogorov‐Smirnov test (procedure UNIVARIATE). *P* ≤ .05 was defined as TYPE I error threshold.

## 3 RESULTS

### 3.1 Study samples

Seven European short hair cats were included. Four cats were intact females, and 3 were intact males. The median age was 5.5 years (range, 4‐7 years). The median weight was 4.5 kg (range, 3.5‐5.3 kg).

All 7 cats appeared bright and active throughout the study period. No vomiting or diarrhea was observed on any day by the caregivers. Body weights did not change significantly over time (*P* = .71), and none of the physical examinations detected any abnormalities.

### 3.2 Lipase activity and PLI concentration

Median baseline (d1) lipase activity was 22 U/L (range, 14‐52 U/L) and median baseline (d1) PLI was 3.2 μg/L (2.3‐15.7 μg/L). One male cat (red dots in all figures) had increased lipase activity (52 U/L) and PLI concentration (15.7 μg/L) before first administration of prednisolone. Two other cats had baseline PLI concentrations above the previous (>3.5 μg/L) and new (4.4 μg/L) RI (4.6 and 5.4 μg/L). Median lipase activities and PLI concentrations without the cat with increased baseline values were 22 U/L (14‐26 U/L) and 3 μg/L (2.3‐5.4 μg/L).

**FIGURE 1:**
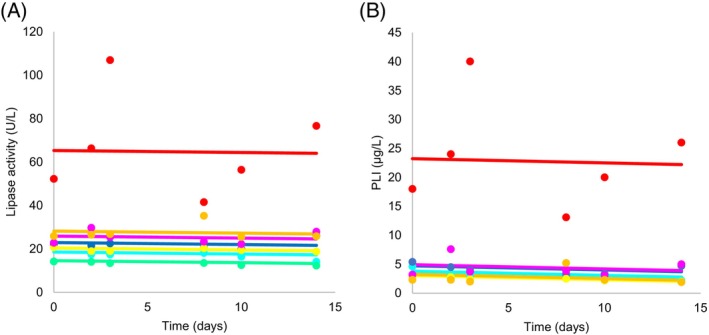
(A) Random intercept mixed model analysis of the response of lipase activity over time in cats administered prednisolone from day 1 to day 7. There was no significant change in lipase activity over time (*P* = .2). Statistical significance was set at *P* < .05. (B) Random intercept mixed model analysis of the response of pancreatic lipase immunoreactivity (PLI) concentration over time in cats administered prednisolone from day 1 to day 7. There was no significant change in PLI concentration (*P* = .29) over time. Statistical significance was set at *P* < .05.

Comparing lipase activity and PLI concentrations over time, there was no significant change in lipase activity (*P* = .2) and PLI concentration (*P* = .29; Figure 1a,b). This was the same when the cat with increased lipase activity/PLI concentration was excluded from the analysis (*P* = .12, lipase activity; *P* = .07, PLI).

**FIGURE 2:**
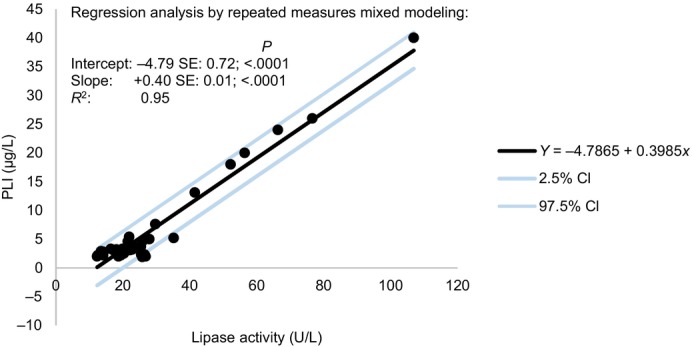
Repeated‐measures mixed model linear regression analysis on the relationship of serum lipase activity and pancreatic lipase immunoreactivity (PLI) concentration in cats administered prednisolone over 7 consecutive days. We followed the statistical approach proposed by Shan et al.^17^ CI, confidence interval.

The results of a repeated‐measures mixed model linear regression analysis revealed linear dependency of the 2 lipase assays (*R* ^2^ = 0.95, *P* < .0001; Figure 2). Increased lipase activity values coincided with increased PLI concentrations.

### 3.3 Further results of 1 cat with abnormally high lipase activity and PLI concentration

**FIGURE 3:**
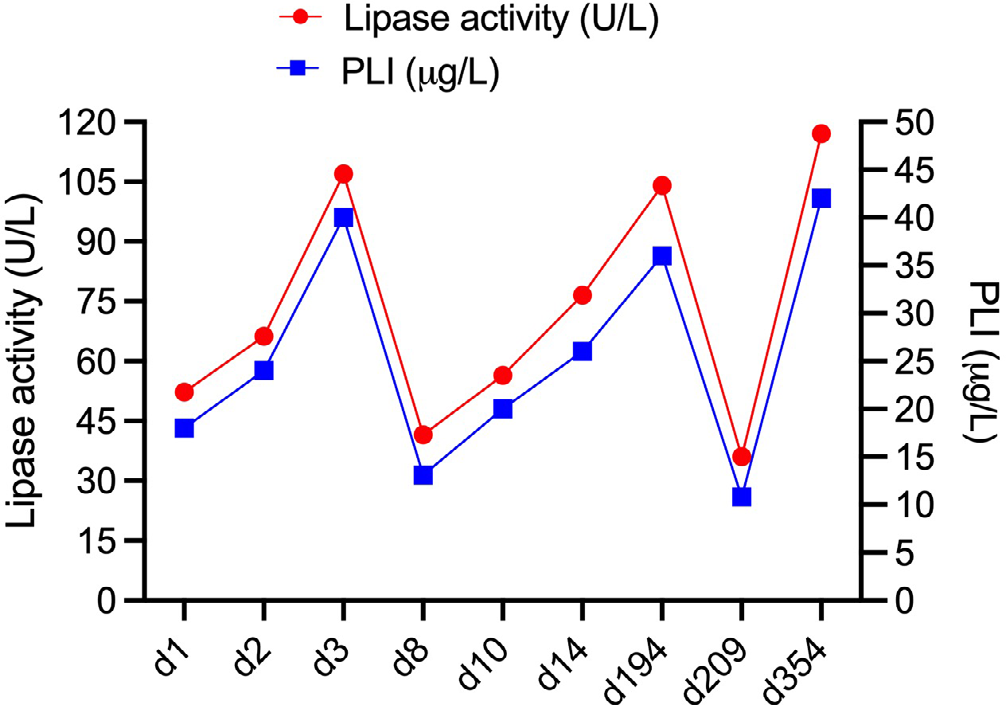
Lipase activities and pancreatic lipase immunoreactivity (PLI) concentrations in 1 cat with continuously increased lipase results. The cat remained clinically healthy throughout the observation period. d, day.

The cat with increased baseline lipase values continued to have elevated lipase activities and PLI concentrations fluctuating over time in a clearly abnormal range throughout the 14‐day study period. Magnitude and nature of change were very similar for both assay results. The cat remained clinically healthy albeit there was a slight decrease (3.9%) in body weight from d1 to d14. The cat remained clinically healthy when rechecked 3 more times over the span of 1 year. Its body weight kept oscillating around the baseline weight and had increased by 300 g in the last 6 months. Lipase activity and PLI concentration remained clearly increased during additional follow‐up examinations (Figure 3). Again, the magnitude and nature of change were very similar for both lipase assays. The cat's pancreas was also examined ultrasonographically 3 times by 2 board‐certified radiologists. Upon first ultrasonographic examination on d10, all parts of the pancreas could be well visualized and pancreatic size, contours, and ducts were deemed normal. The parenchyma of the splenic limb and corpus was slightly hypoechoic. The diagnosis was a possible mild pancreatitis. However, the radiologist made a remark that the cat as a relatively slender large male cat had little mesenteric fat, and this may have contributed to the hypoechogenicity of the splenic limb and corpus. Upon second evaluation on d209 after study completion, the pancreas appeared mildly enlarged (corpus, left branch), its shape was rounded, the surface mildly irregular, and the echogenicity mildly reduced. The adjacent mesentery was slightly hyperechoic. In addition, a thickened small intestinal muscularis layer, mildly irregular dilatation of the extrahepatic bile ducts, and mild jejunal lymph node enlargement was present. Ultrasonographic findings were interpreted as chronic active pancreatitis, chronic enteropathy, regional lymphadenopathy, and suspicion of chronic cholangitis. Upon third evaluation, 345 days after study completion, ultrasonographic findings in the pancreas, small intestine, lymph nodes, and bile ducts could be reproduced.

### 3.4 Triglyceride and cholesterol concentration

**FIGURE 4:**
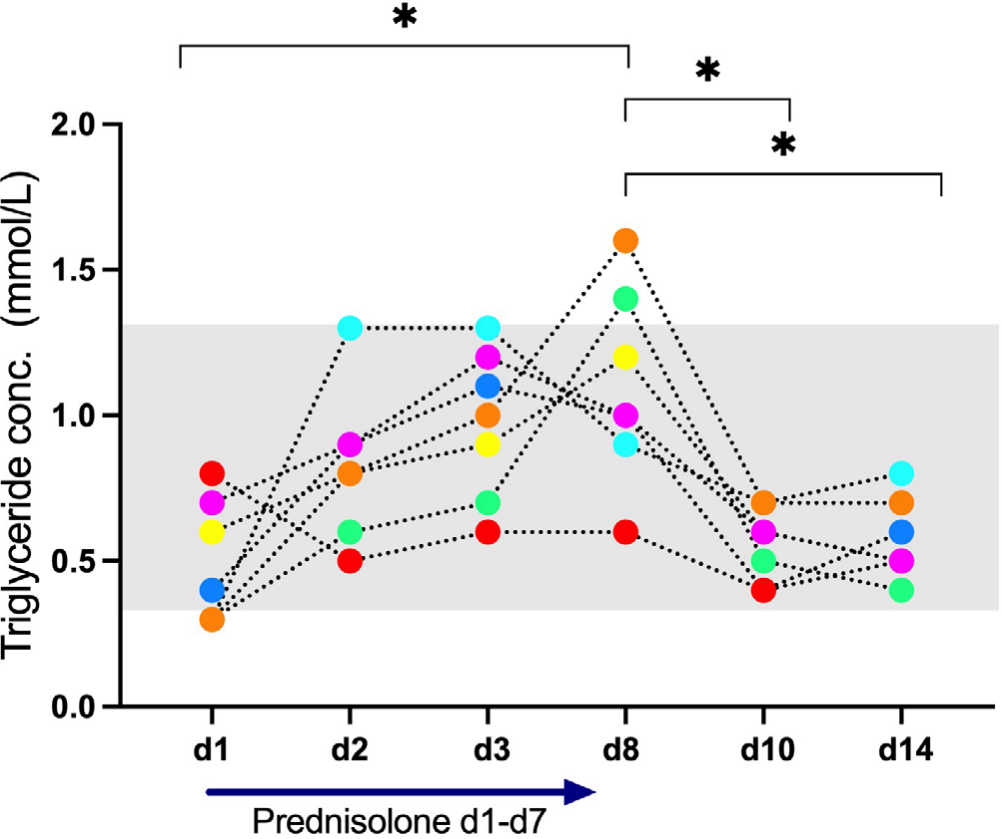
Triglyceride concentration (conc.) measured in 7 clinically healthy cats. Prednisolone was given daily from day (d) 1 to d7. The gray shaded area symbolizes the reference interval.

**FIGURE 5:**
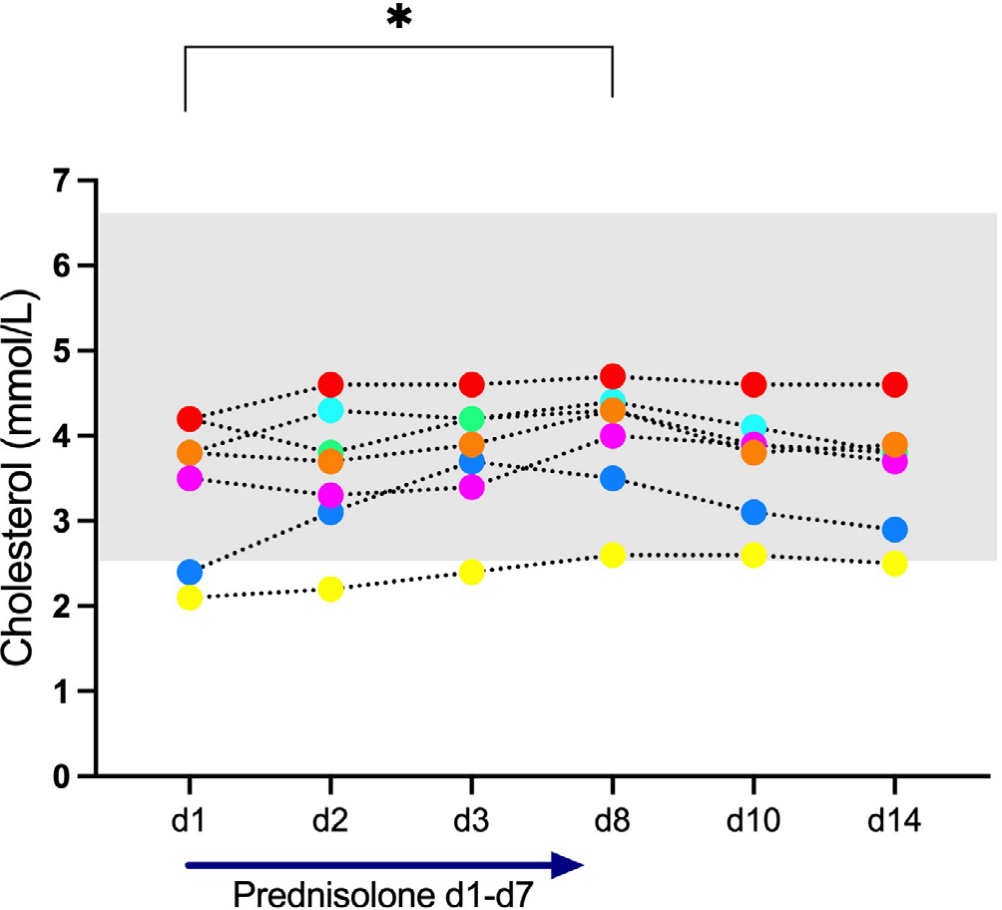
Cholesterol concentration measured in 7 clinically healthy cats. Prednisolone was given daily from day (d) 1 to d7. The gray shaded area symbolizes the reference interval. Friedman test; statistical significance set at *P* < .05.

Triglyceride concentrations increased significantly under prednisolone and decreased again significantly when prednisolone was discontinued (Figure 4). All but 2 results were within RI. Similarly, cholesterol concentrations were also significantly increased at d8. Again, changes remained within RI (Figure 5). No significant correlations were found between either triglyceride or cholesterol concentrations and both lipase assay results. Triglyceride and cholesterol concentrations remained within RI in the cat with continuously increased lipase activities and PLI concentrations.

### 3.5 SAA concentration

**FIGURE 6:**
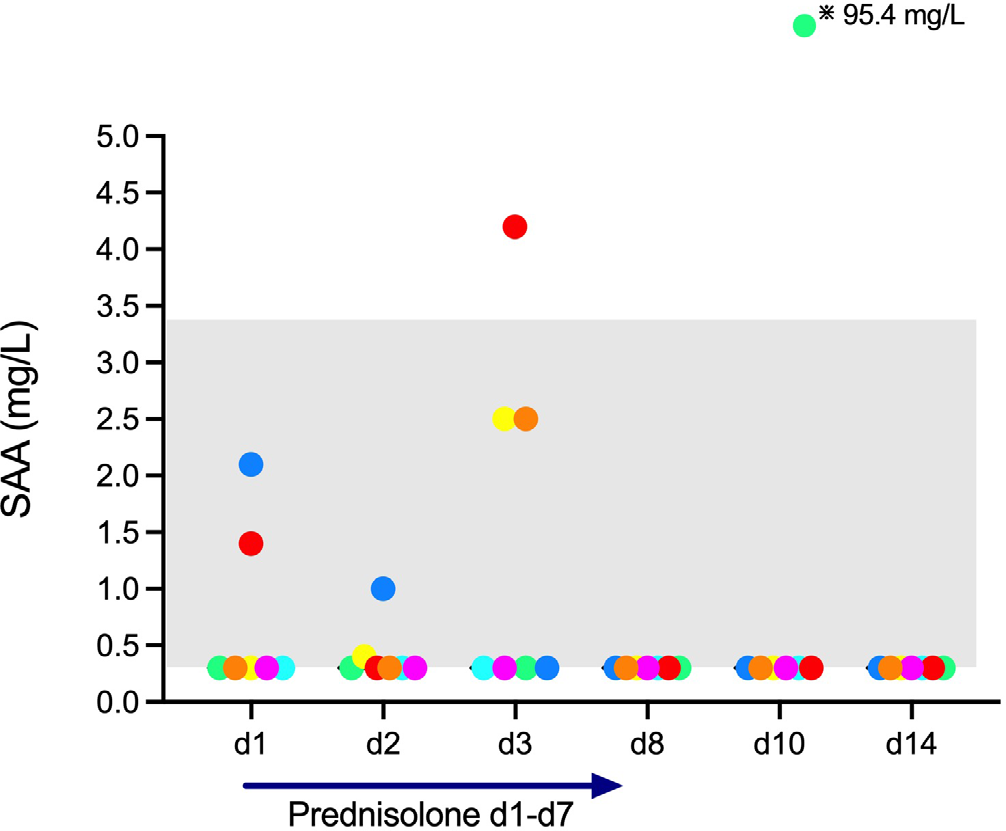
Serum amyloid A (SAA) concentration measured in 7 clinically healthy cats. Prednisolone was given daily from day (d) 1 to d7.The gray shaded area symbolizes the reference interval. Serum amyloid A concentrations were measurable 8 out of 42 times in 5 cats; 34 measurements were <0.3 mg/L.

Serum amyloid A concentrations were measurable 8 out of 42 times in 5 cats during the study (Figure 6). All but 2 SAA results were within RI. The cat with increased lipase activities and PLI concentrations had measurable SAA concentration on 2 time points: 1.4 mg/L at baseline (d1) and 4.1 mg/L at d3.

## 4 DISCUSSION

We investigated the influence of prednisolone administration on serum lipase measured as an activity and concentration in healthy cats. Short‐term administration of anti‐inflammatory doses of prednisolone did not significantly affect lipase activity or PLI concentration in clinically healthy cats.

We believe these results are relevant as prednisolone is frequently used in cats with a variety of inflammatory diseases, and prednisolone is also increasingly discussed as a treatment regime for chronic pancreatitis in cats.^3^ Also, cholangitis and inflammatory bowel disease are often coexisting with pancreatitis,^18^, ^19^, ^20^ and anti‐inflammatory doses of prednisolone are commonly used when treating these conditions. Knowing that prednisolone does not per se affect serum lipase activity and PLI concentration in cats is helpful because an increased lipase in a nonspecifically ill cat would inevitably be equated with the presence of pancreatitis in daily practice.

Our results support the findings of studies in dogs. In a prospective study, 17 dogs were treated with mostly anti‐inflammatory doses of prednisolone for 3 to 4 weeks, and only minimal lipase activity increases (DGGR‐based assay, Randox) within RI were observed.^21^ In another prospective study, 10 dogs were treated with anti‐inflammatory doses of prednisolone over 10 days and neither PLI concentrations nor lipase activities (1,2 diglyceride–based assay, Abbott) increased significantly.^22^

Even if it was not the main goal of the study, we could also compare both lipase assays. We found a significant positive correlation between lipase activity and PLI concentration. This appeared lower compared with what has been previously published when comparing both assays in cats with suspicion of pancreatitis.^6^, ^8^ We assume this is because lipases were measured in clinically healthy cats. Figure 2 shows that higher variation was observed with low values within RI.

The concern that extrapancreatic lipases hydrolyze the substrate DGGR in healthy cats, and thus contributing to lipase activity in healthy cats without pancreatitis was raised recently.^23^ In that study, 6 healthy neutered cats were given heparin IV and lipase activities (Diazyme Laboratories, Inc.) were measured 10, 20, 30, 60, and 120 minutes after IV heparin administration.^23^ There was 1 single significant increase in lipase activities at 10 minutes post IV heparin compared with baseline. Because no RI for the lipase activity assay was given, it remains unclear whether all results were also within RI.^23^

We believe that the debate about possible influences of extrapancreatic lipases on lipase activity results within RI in healthy cats is not helpful as changes within RI are clinically irrelevant. It should be noted that the diagnosis of acute pancreatitis in people is based on lipase activity^4^ 3 times greater than the upper RI limit, the presence of compatible clinical signs, imaging results, or both.^24^ Comeasurement of extrapancreatic lipases is considered negligible in this scenario.^24^

Interpretation of PLI results has recently changed without any modification being made to the assay.^14^ The upper RI limit was increased from 3.5 to 4.4 μg/L, and PLI results that used to be “consistent” with pancreatitis until mid‐2023 are now regarded to be in an equivocal range. These new calculations based on a larger number of healthy cats resulted in a decision threshold ≥8.8 μg/L that was again denoted as “consistent with pancreatitis.”^14^ Those higher cutoffs fit the observation that increased lipase concentrations are found in clinically normal cats, although it remains unclear whether these cats ultimately have subclinical pancreatitis or not. These new PLI cutoffs are in contrast to the clinical assessment of disease severity markers in 45 cats with suspected pancreatitis in another recent study, where 32/45 (71%) cats with suspected pancreatitis had PLI concentrations <3.5 μg/L.^25^

The new PLI cutoffs do not affect our results and conclusions, as all PLI results >3.5 μg/L at baseline and during prednisolone treatment were also >4.4 μg/L, and the 1 cat with increased lipase values had much higher results.

Corticosteroids upregulate hormone‐sensitive lipase and lipoprotein lipase thus promoting lipolysis, and prednisolone causes hyperlipidemia in healthy cats.^11^, ^12^ Hypercholesterolemia is also the most commonly reported biochemical abnormality in cats with pancreatitis^26^ and hypertriglyceridemia is reported in 57/104 (55%) cats with pancreatitis.^7^ But it remains unclear if hyperlipidemia is a cause or a consequence of pancreatitis in cats. In humans, hyperlipidemia has been implicated as a possible cause of pancreatitis.^27^, ^28^ It has been postulated that pancreatic lipase might break down triglycerides to fatty acids within the pancreas resulting in acinar damage.^28^ We comeasured cholesterol and triglyceride concentrations together with lipases and although significant increases were found during prednisolone administration, changes were mostly within RI. We also found no correlation among cholesterol, triglycerides, and either lipase activity or PLI concentration. Probably, more pronounced hyperlipidemia is needed to examine if there is an association among cholesterol, triglycerides, and lipase activity/PLI concentrations.

One of the 7 cats had continuously increased lipase activities and PLI concentrations. An influence of prednisolone on lipase results seems unlikely considering the fluctuating pattern during the study period and also over the span of 1 year (Figure 3). Combined with US findings, a diagnosis of subclinical chronic pancreatitis seems valid. Prednisolone has been discussed as a treatment for chronic pancreatitis in cats.^3^ Median PLI concentrations increased in a recent abstract (Wu et al., Abstract GI28 ACVIM Forum 2022, *J Vet Intern Med*, 36:2427‐2428) when cats with chronic pancreatitis were treated with higher doses of prednisolone (4 mg/kg for 5 days, then 2 mg/kg), but it was not determined if this increase in median PLI concentrations was significant. Lipase activities did not decrease in the cat with pancreatitis when given prednisolone.

We used anti‐inflammatory doses of prednisolone in our study, as this is the most commonly used dosage when treating inflammatory gastrointestinal or hepatobiliary disease in cats in our hospital. It is possible that higher doses and longer term administration may have led to different results. When dogs were treated with immunosuppressive dosages of prednisolone or dexamethasone, PLI and lipase activity (substrate olive oil, Sigma Chemical) increases into ranges considered diagnostic for pancreatitis have been observed.^16^, ^29^

Although steroid‐induced pancreatitis is exceedingly rare in humans and has not been reported in cats so far, we had decided to concurrently measure SAA concentrations as a sensitive marker for systemic inflammation in case some cats would react with increased lipase results to prednisolone treatment and the clinical picture would be equivocal. Increased SAA concentrations occur in cats with pancreatitis^30^, ^31^ and could be a useful tool for monitoring disease severity.^31^ Serum amyloid A concentrations were normal at d1, minimally increased in 3 cats at d3, and below the detection limit for all other time points in the cat with increased lipase activities and PLI concentrations, which together with the absence of clinical signs, makes a chronic pancreatitis more likely. Correlation analysis between SAA and lipase measurements was not possible because 34/42 SAA results were < 0.3 mg/L. One cat had a sudden and sharp increase in SAA concentration (95.4 mg/L) at d10 (2 days after discontinuation of prednisolone), which was preceded and followed by unmeasurably low SAA concentrations. We speculate this was secondary to interactions among cats resulting in scratches not detected during daily physical examination. Because all clinical variables, as well as observations of the caregivers and all other laboratory results, were normal in this cat.

Our study had some limitations. The study sample was comparatively small; still a power analysis indicated that 6 cats were sufficient to detect a statistically significant difference.

Food was not withheld for laboratory examination, and this might have affected laboratory results. However, a recent study evaluating the effect of prior food intake on standard laboratory values in dogs found no increase in lipase activity (substrate 1,2 diglyceride, Beckman Coulter).^32^ We measured lipase 24 hours after the previous prednisolone administration. Lipase half‐life time is unknown in cats. Given the fact that oral prednisolone reaches maximum concentration after approximately 1 hour in cats and has a half‐life time of elimination of 0.66 hour,^33^, ^34^ we cannot exclude that measuring lipase 4 to 6 hours after drug administration would have led to different results. However, the duration of action of prednisolone is believed to be between 12 and 26 hours.^34^ Still, we believe this aspect is of minor clinical relevance because there are always variable time differences between blood sampling and tablet administration in daily practice.

## 5 CONCLUSION

Short‐term administration of anti‐inflammatory doses of prednisolone does not affect lipase activity and PLI concentration in healthy cats.

## CONFLICT OF INTEREST DECLARATION

Authors declare no conflict of interest.

## OFF‐LABEL ANTIMICROBIAL DECLARATION

Authors declare no off‐label use of antimicrobials.

## INSTITUTIONAL ANIMAL CARE AND USE COMMITTEE (IACUC) OR OTHER APPROVAL DECLARATION

Approved by the cantonal veterinary office of Zurich, ZH195/2021.

## ETHICS APPROVAL DECLARATION

Authors declare human ethics approval was not needed for this study.

## Supporting information


**Figure S1.** Daily recorded body weights (kg) in 7 clinically healthy cats. Prednisolone was given daily from day 1 to day 7.

**Figure S2.** Glucose concentration measured in 7 clinically healthy cats. Prednisolone was given daily from day 1 to day 7. The grey shaded area symbolizes the reference interval.
