# Supplementary figures and images for: Effects of prednisolone on 1,2‐O‐dilauryl‐rac‐glycero glutaric acid‐(60‐methylresorufin) ester‐lipase activity and pancreatic lipase immunoreactivity in healthy cats
Source: J Vet Intern Med. 2024 Mar 14;38(3):1370–6. doi: 10.1111/jvim.17042 (PMC11099763; doi:10.1111/jvim.17042)

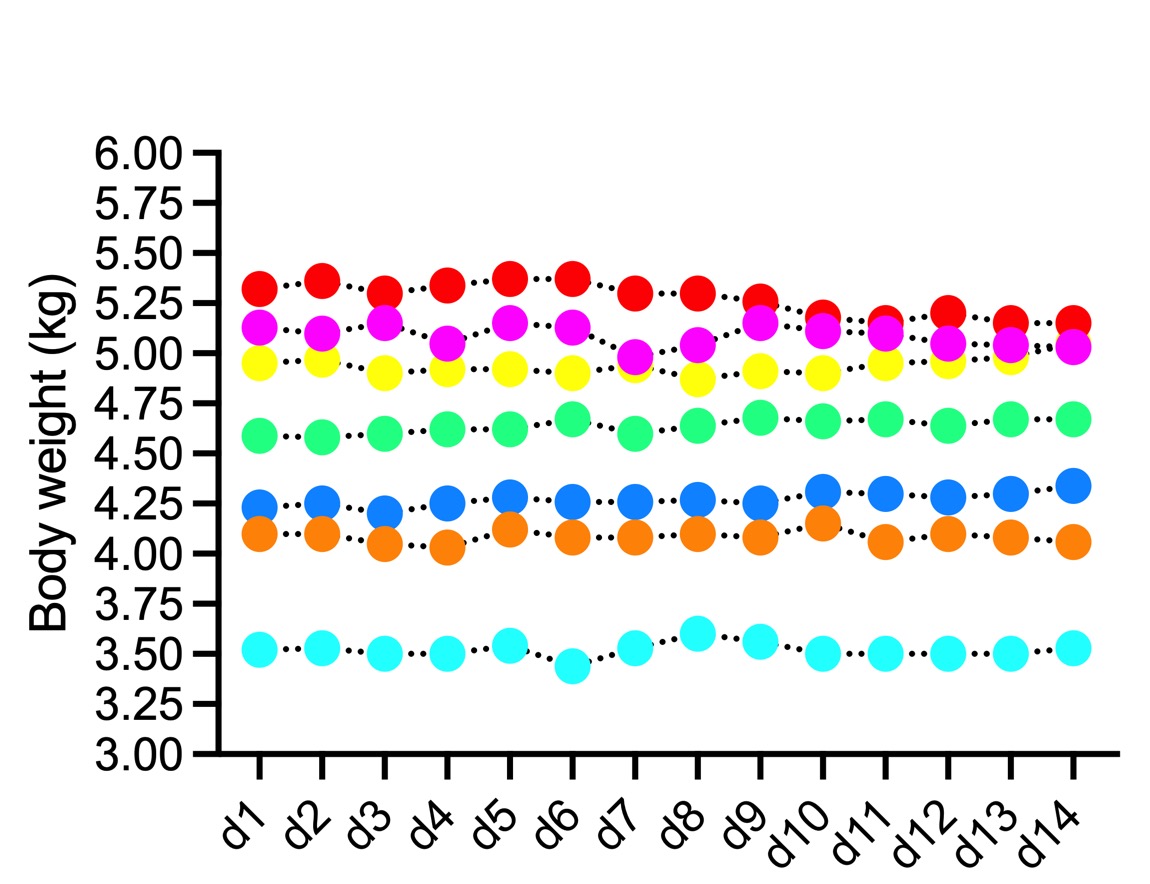

Supplement: Supplementary file 1 — Figure S1. Daily recorded body weights (kg) in 7 clinically healthy cats. Prednisolone was given daily from day 1 to day 7. [file JVIM-38-1370-s002.jpg]

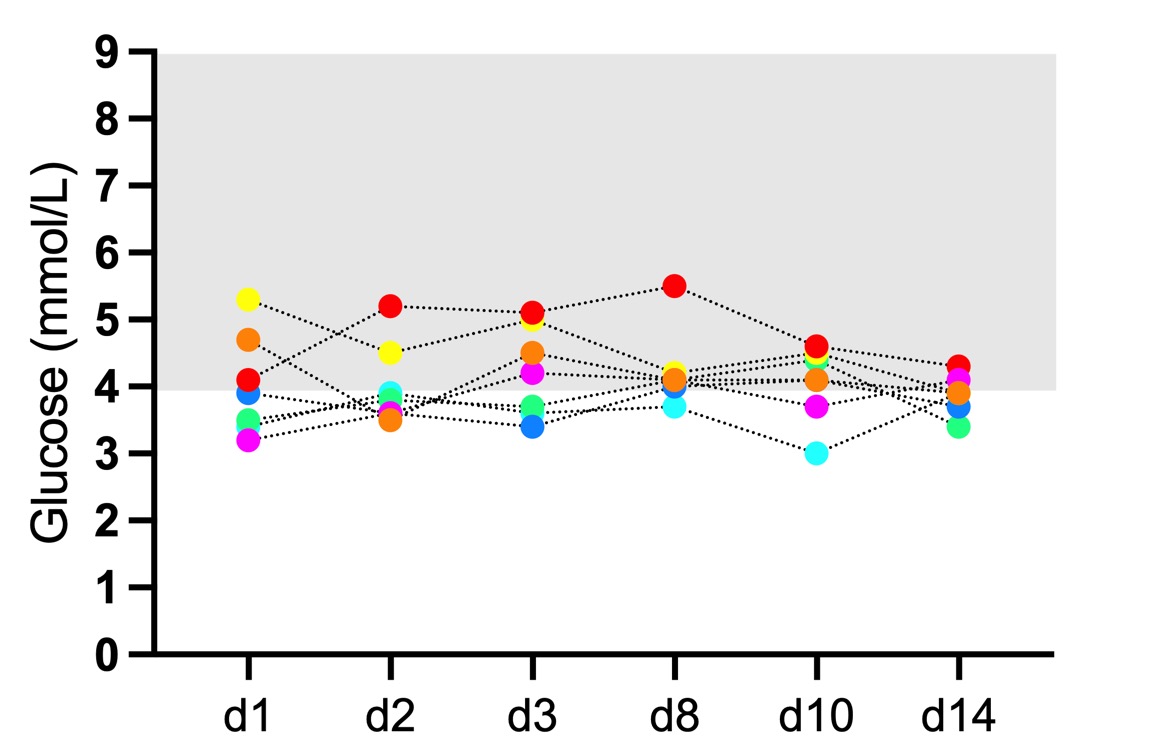

Supplement: Supplementary file 2 — Figure S2. Glucose concentration measured in 7 clinically healthy cats. Prednisolone was given daily from day 1 to day 7. The grey shaded area symbolizes the reference interval. [file JVIM-38-1370-s001.jpg]
